# Identification of a competing endogenous RNA axis related to gastric cancer

**DOI:** 10.18632/aging.103926

**Published:** 2020-10-20

**Authors:** Fuqiang Zu, Hezhou Han, Weiwei Sheng, Jian Sun, Hui Zang, Yu Liang, Qingfeng Liu

**Affiliations:** 1 Department of General Surgery, The People’s Hospital of China Medical University, Shenyang 110016, Liaoning, China; 2 Central Laboratory of General Surgery, Shengjing Hospital of China Medical University, Shenyang 110004, Liaoning, China; 3 Department of Gastrointestinal and Hernia Surgery, First Affiliated Hospital of China Medical University, Shenyang 110001, Liaoning, China

**Keywords:** integrated analysis, competing endogenous RNA, gastric cancer, progression

## Abstract

Competing endogenous RNA (ceRNA) pathways play pivotal roles in the formation and progression of gastric cancer (GC). Employing multi-omics analysis, we sought to identify a ceRNA network associated with GC progression. We analyzed3Gene Expression Omnibus datasets as well as data from The Cancer Genome Atlas to identify genes that were differentially expressed in GC tissues. A total of 84 upregulated genes and 106 downregulated genes were found. Enrichment analysis indicated that some pathways were strongly linked with tumor formation and progression. We also screened hub genes to establish a lncRNA-miRNA-mRNA network. We ultimately identified 8 hub genes, 6 key miRNAs and 4 key lncRNAs that interact within a common ceRNA network. Correlation analysis and in vitro experiments were conducted to verify the regulatory effect of the ceRNA network in GC. A knockdown assay confirmed that the *DLGAP1-AS1/miR-203a-3p/THBS2* axis is a ceRNA network involved in GC progression. In this study, we elucidated the role of the *DLGAP1-AS1/miR-203a-3p/THBS2* ceRNA network in the progression of GC. These molecules maybe evaluated as therapeutic targets and prognostic biomarkers for GC.

## INTRODUCTION

Although there has been a decline in the incidence rate, gastric cancer remains the fourth most common malignancy and the second most deadly neoplasm globally. Over a million newly diagnosed cases annually, resulting in 783,000 deaths in 2018 [1]. Owing to a lack of specific symptoms in the early stage, the majority of patients with GC are diagnosed at advanced stages, which reduces the chances for a radical resection [2].

Non-coding RNA (ncRNA) does not directly code functional protein, but it is abundant in the human genome. Accumulating evidence has indicated that ncRNAs, including microRNA (miRNA), long noncoding RNA (lncRNA) and circular RNA (circRNA), play an important role in oncogenesis and tumor progression of various cancers such as GC [3–5]. ncRNA can combine with protein-coding mRNAs and regulate gene expression at the transcriptional and post-transcriptional levels [6]. Moreover, multiple RNAs can interact with each other via miRNA response elements and assemble as a competing endogenous RNA (ceRNA) network [7]. In this network, lncRNA can act as a "sponge" to absorb and bind miRNA, thereby weakening its binding ability to mRNA. Emerging data have suggested that ceRNA networks play a pivotal role in progression and metastasis in breast cancer, ovarian cancer and GC [8–10].

In our work, we determined which genes are differentially expressed (DEGs) in GC tissues compared to normal tissues. Datasets for analysis were obtained from the Gene Expression Omnibus (GEO) and The Cancer Genome Atlas (TCGA) databases. Protein-protein interaction (PPI) networks were constructed by a string database and we distinguished the top 10 hub genes according to their degrees score. Hub genes refer to genes with high connectivity. These genes play a crucial role in the biological function of another gene that is a functional target. According to the ceRNA hypothesis, lncRNA can diminish miRNA activity via adsorptive action. Therefore, a qualified candidate lncRNA should be negatively linked with miRNA expression and positively correlated with the mRNA level at the same time [7, 11]. Following this hypothesis, we predicted gene-related upstream miRNA via the miRecords database, and miRNA-linked upstream lncRNA through the miRNet dataset. The prognostic properties of the chosen RNAs were assessed both in bioinformatics databases and using qRT-PCR methods. Ultimately, a promising ceRNA regulatory network related to the progression of GC was successfully identified.

## RESULTS

### DEG identification

**Figure 1:**
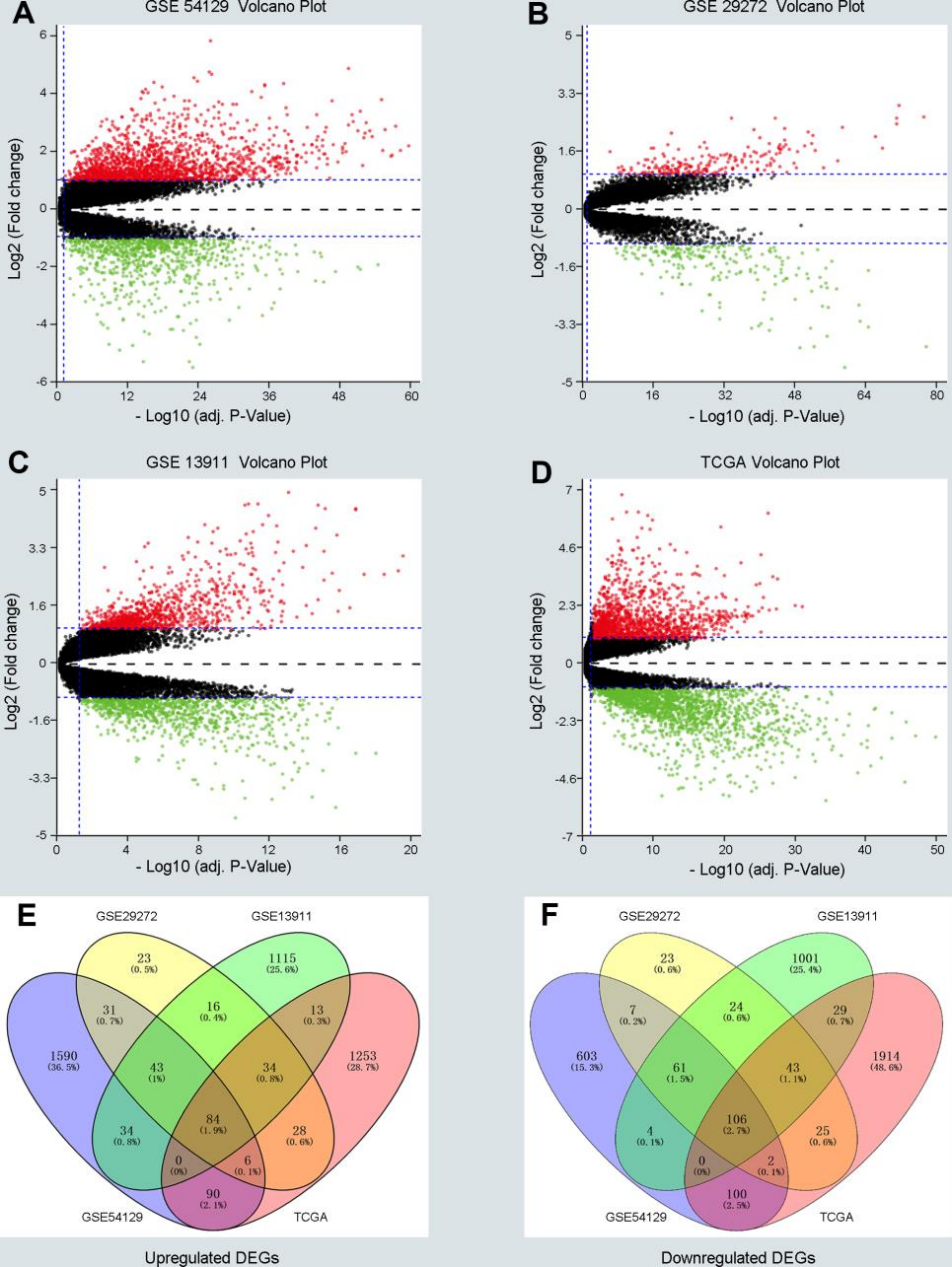
**Screening differentially expressed genes (DEGs) between gastric cancer (GC) and normal samples in three GEO datasets and TCGA database.** (**A**–**D**) The volcano plots of DEGs in GSE54129, GSE29272, GSE13911 and TCGA datasets with thresholds of |log2FC| > 1, adjust P value < 0.05. The red dots and green dots represent the upregulated and downregulated DEGs separately. The black dots mean no significantly different genes. (**E**, **F**) The intersection of upregulated DEGs and downregulated DEGs in four datasets, respectively.

Three GEO (GSE54129, GSE29272, GSE13911) and one TCGA dataset were enrolled in a training group comprising663 cancer samples and 223 normal cases. Dataset GSE54129 contained111 GC samples and 21 non-tumor samples; dataset GSE29272 contained 134 GC samples and 134 non-tumor samples; dataset GSE13911 contained 38 GC tissues and 31 non-tumor samples; and the TGCA dataset contained 380 GC tissues and 37 non-tumor samples. DEGs in the training group were identified and are displayed in the volcano plot in Figure 1 with thresholds of |log2FC| >1 and a *P* value < 0.05 (Figure 1A–1D). As depicted in Venn plots (Figure 1E, 1F), we integrated commonly expressed genes that intersected in the training group, and we successfully identified 84 upregulated and 106 downregulated DEGs. The details of these DEGs, which were chosen for the following analysis, can be found in Supplementary Table 1 (Supplementary Table 1).

### Enrichment analysis of DEGs

**Figure 2:**
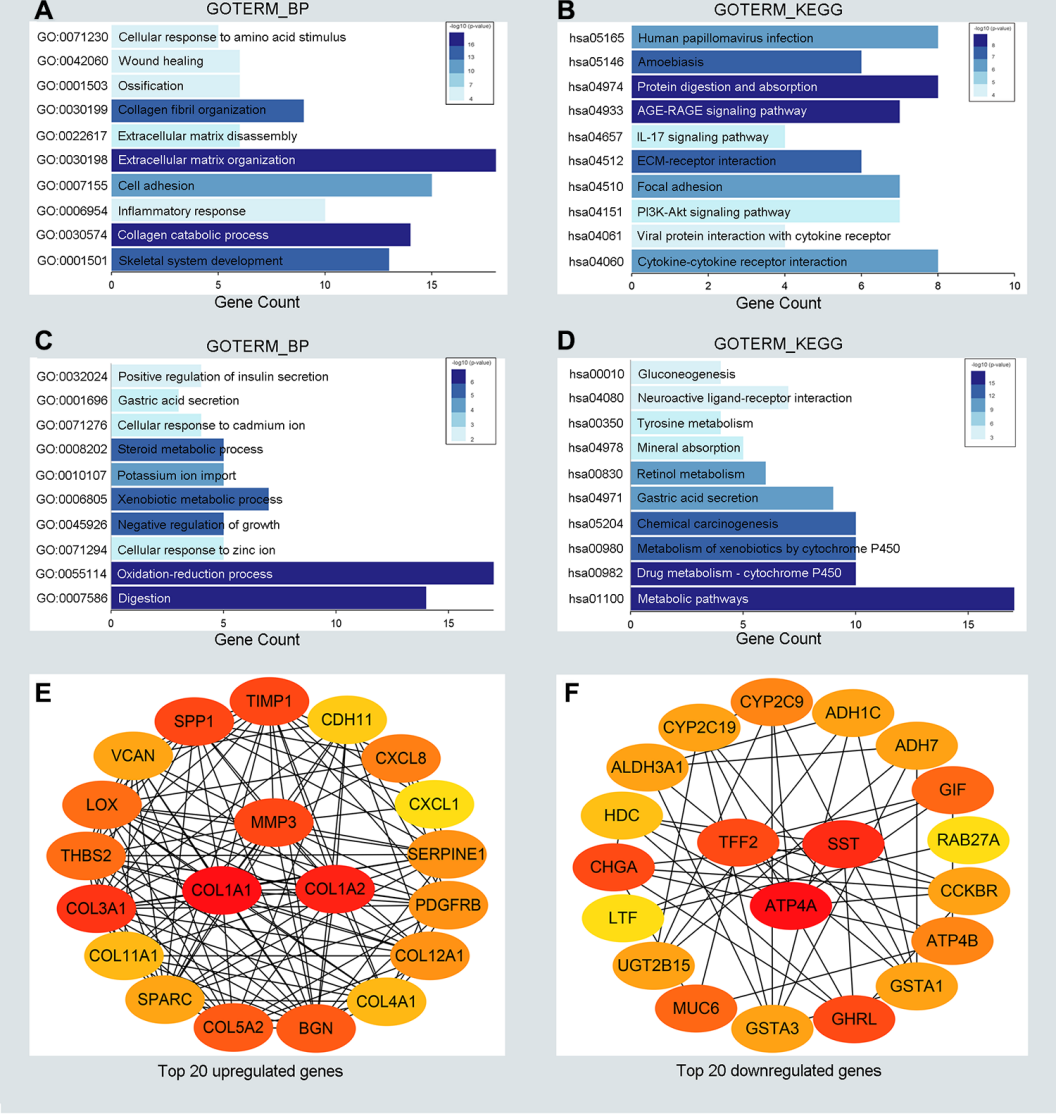
**Functional enrichment analysis for the significant DEGs and identification of hub genes.** (**A**, **C**) The top ten enriched biological processes of the significantly upregulated DEGs and downregulated DEGs, respectively. (**B**, **D**) The top ten enriched KEGG pathways of the significantly upregulated DEGs and downregulated DEGs, respectively. (**E**, **F**) The top 20 hub genes of the significantly upregulated DEGs and downregulated DEGs separately.

To detect potential biological functions of upregulated and downregulated DEGs, gene ontology (GO) enrichment analysis and KEGG pathway analysis were conducted. As depicted in Figure 2A, 2B, upregulated genes were mainly enriched in the extracellular matrix organization and cell adhesion process (Figure 2A). Additionally, several cancer-related pathways were detected in the KEGG pathway analysis, such as the *PI3K-AKT* signaling pathway and cytokine receptor interaction pathway (Figure 2B). Downregulated genes were mainly enriched in the oxidation-reduction process and xenobiotic metabolic process (Figure 2C). Genes associated mainly with the KEGG pathway were those involved in cytochrome P450 metabolism and other metabolic pathways (Figure 2D). GO enrichment analysis of the molecular function and cellular component of the DEGs are depicted in Supplementary Figure 1. In generally, the results from functional enrichment analysis were tightly linked with GC.

### Screening and validation of hub genes

**Figure 3:**
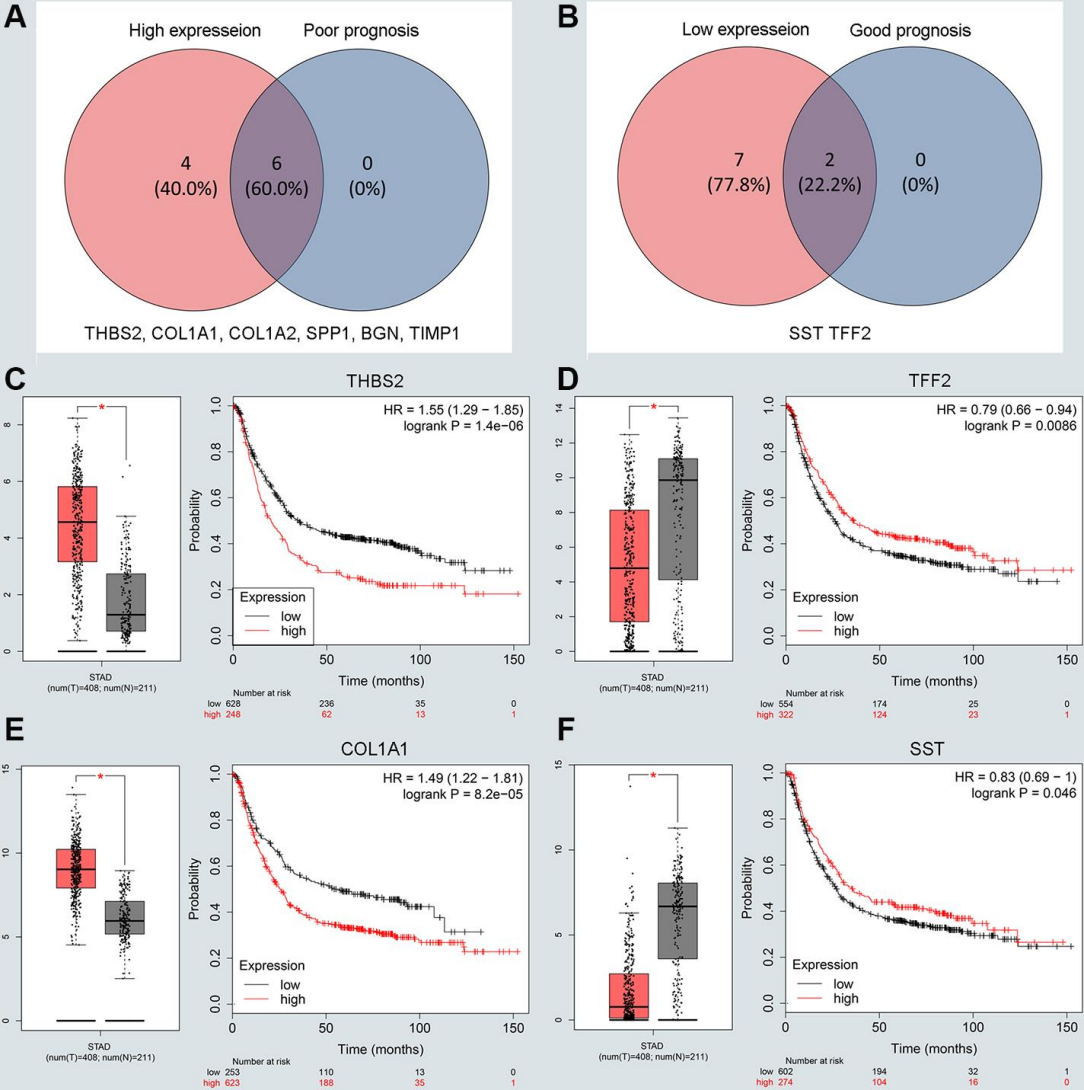
**Screening and validating the expression roles and prognosis values of key genes in GC.** (**A**) Screening the key genes with high expression and dismal prognosis values in upregulated hub genes. (**B**) Screening the key genes with low expression and good prognosis values in downregulated hub genes. (**C**–**F**) Validating expression roles and prognosis values of key genes in hub genes using GEPIA and Kaplan–Meier plotter databases.

To understand the interaction between the upregulated DEG group and the downregulated DEG group separately, a PPI network was constructed. We calculated the node degree of the PPI network using cytoHubba tools from Cytoscape software and classified the top 20 hub genes into two groups (Figure 2E, 2F). Next, to improve our result reliability, we verified the top 10 hub genes in the validation group. The GSE27342 dataset contained 80 GC samples and 80 normal samples; theGSE37023 dataset contained112 tumor samples and 39 normal samples; and theGSE65801 dataset contained 32 cancer samples and 32 normal samples. All the hub genes were involved in the validation group (Supplementary Figure 2A, 2B). Subsequently, GEPIA and Kaplan Meier (KM) plot database was performed to assess each hub gene’s expression and its relationship to prognosis. In the upregulated hub gene group, 6 genes (*COL1A1, COL1A2, TIMP1, SPP1*, *BGN, and THBS2*) were not only significantly upregulated in GC but also strongly correlated with poor GC prognosis (Figure 3A, 3C and 3E). As for downregulated hub genes, two genes (*SST, TFF2*) were expressed at a low level in GC and were correlated with a favorable GC prognosis (Figure 3B, 3D and 3F). Other candidate hub genes, which were chosen for the following analysis, are shown in Supplementary Figure 2C–2F).

### Identification and validation of upstream miRNA

Based on the results from candidate hub genes, we identified the upstream miRNA of those genes through the miRNA-target interactions database, miRecords. Only miRNAs that appeared at least 3 times were considered as candidate miRNAs. A total of 136 miRNAs were predicted to regulate 6 hub genes. Only 13 miRNAs that were identified as candidate miRNAs (Supplementary Table 2). Additionally, the starBase platform was applied to validate the expression role of candidate miRNAs, and the KM plot database was used to verify the prognostic value of candidate miRNAs. As shown in Supplementary Figures 3 and 4, we confirmed that 6 miRNAs are related to upregulated hub genes (*hsa-miR-203a-3p, hsa-miR-204-5p, hsa-miR-26a-5p, hsa-miR-339-5p, hsa-miR-1225-3p* and *hsa-miR-378a*). Not only was the expression of these genes downregulated in GC, but they were also linked with poor prognosis in the disease. However, for downregulated hub genes, we found only one miRNA (*hsa-mir-9-5p*) that was upregulated in GC and associated with a favorable prognosis. All qualified miRNAs were selected for further tests.

### Prediction and validation of upstream lncRNA

**Figure 4:**
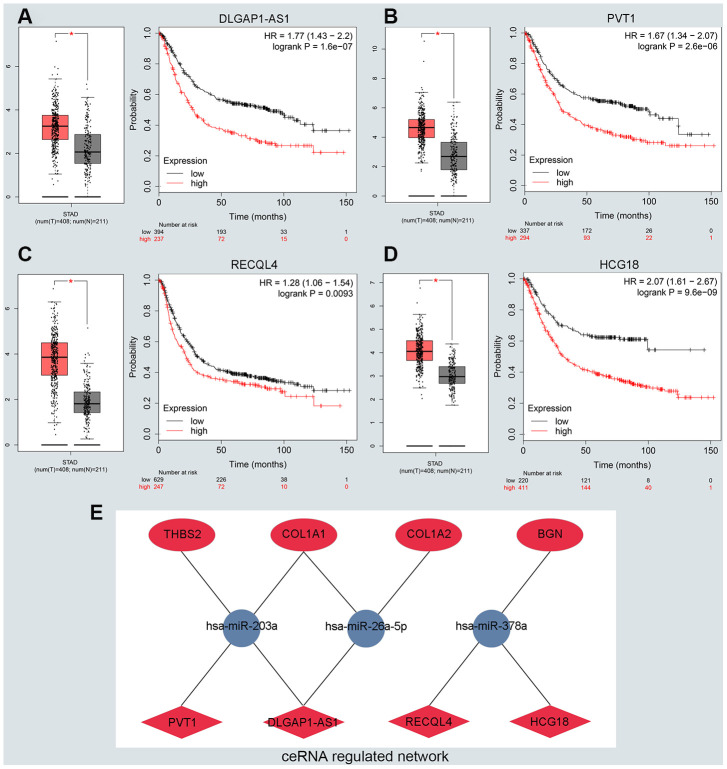
**Identifying the key long noncoding RNA and constructing the ceRNA network in GC.** (**A**–**D**) Validating the expression and prognostic value of four key lncRNAs using GEPIA and Kaplan–Meier plotter databases. (**E**) The potential mRNA-miRNA-lncRNA regulatory network related to GC prognosis. The ellipse, round and diamond shape represents lncRNAs, miRNA and mRNA respectively. Red and blue represent the ups and downs expression, respectively.

To predict upstream lncRNA of candidate miRNAs, we used the miRNet database. According to the prediction in Supplementary Table 3, we identified 139 lncRNAs for 6 downregulated miRNAs and 36 lncRNAs for *miR-9-5p*. Next, GEPIA and the KM plotter database were used to evaluate the expression role and prognostic value of predicted lncRNAs. According to the ceRNA hypothesis motioned above, we screened out 4 eligible lncRNAs (*DLGAP1-AS1, PVT1, RECQL4 and HCG18*) associated with downregulated miRNAs that were both significantly upregulated in GC and correlated with very poor survival (Figure 4A–4D). However, there was no qualified lncRNA that met the expression and prognostic criteria of miR-9-5p. Finally, we identified the eligible lncRNAs, miRNAs and mRNAs that not only satisfied the standards for expression and prognosis but also complied with the ceRNA network hypothesis.

### Construction and verification of the ceRNA network

We constructed a lncRNA-miRNA-mRNA ceRNA network from our previous results. As presented in Figure 4E, there were a total of 8lncRNA-mRNA groups, 5lncRNA-miRNA groups and 5miRNA-mRNA groups. Eligible miRNA has an opposite interaction with mRNA and lncRNA, whereas lncRNA has a positive co-expression relationship with mRNA. We used the starBase platform performed to characterize the co-expression relationships among lncRNA-miRNA and miRNA-mRNA. Ultimately, we established the *DLGAP1-AS1/miR-203a-3p/THBS2* ceRNA pathway, which was not only significantly associated with the prognosis of GC patients but also played pivotal roles in the progression of GC (Supplementary Figure 5A–5C). Other co-expression networks are depicted in Supplementary Figure 5D–5Q.

**Figure 5:**
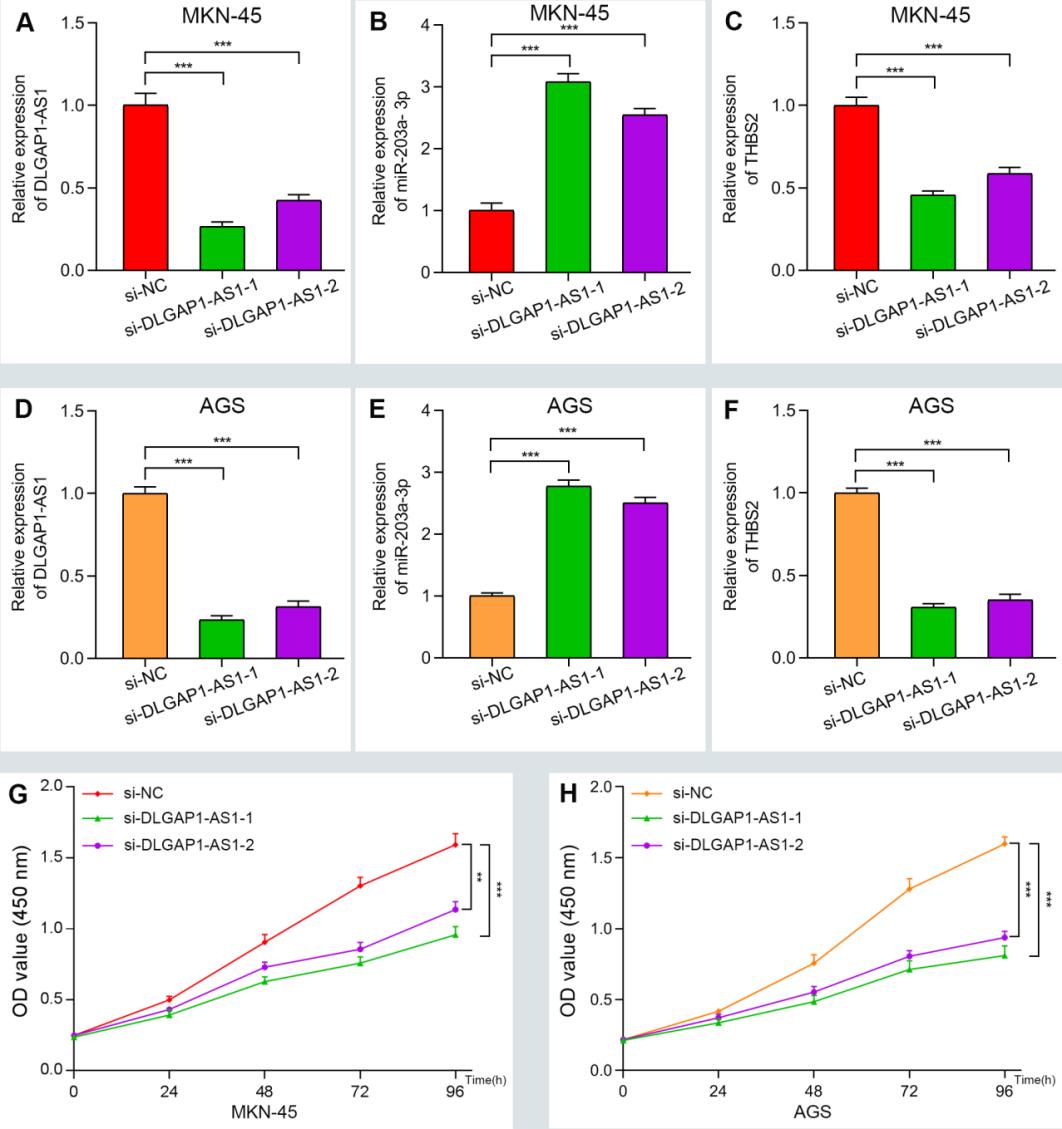
**Verifying the ceRNA network through knockdown and CCK-8 assay.** (**A**–**F**) The relative expression of DLGAP1-AS1 and THBS2 were significantly reduced after silencing DLGAP1-AS1, whereas the relative expression of miR-203a-3p was significantly increased in MKN-45 and AGC cell lines. (**G**–**H**) DLGAP1-AS1 knockdown efficiently suppressed MKN-45 and AGC cell proliferation, respectively. (*P < 0.05, **P < 0.01, ***P < 0.001)

**Figure 6:**
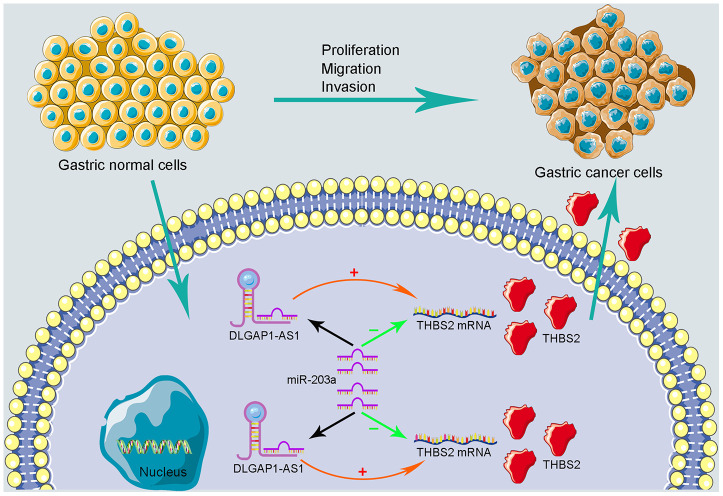
**Schematic representations of DLGAP1-AS1/miR-203a-3p/THBS2 ceRNA pathway and its potential roles in the progression of GC.**

To further evaluate the reliability of our result, we verified the ceRNA network using an in vitro assay. First, the relative expression level of *DLGAP1-AS1* was quantified in 4 GC cell lines (MKN-45, AGS, HGC-27 and MGC-803), as well as and normal gastric cells (GES-1). Our results indicated that MKN-45 and AGS express higher levels of *DLGAP1-AS1* than other cell lines (Supplementary Figure 6A). Therefore, we designed siRNA assay to knockdown the expression of *DLGAP1-AS1* in MKN-45 and AGS cell lines separately and observed the change in relative expression of *DLGAP1-AS1, miR-203a-3p, and THBS2*. The knockdown efficiency was measured by qRT-PCR (Supplementary Figure 6B, 6C). As presented in Figure 5A–5F, the expression levels of *DLGAP1-AS1 and THBS2* were significantly reduced, while the relative expression of *miR-203a-3p* was enhanced after *DLGAP1-AS1* knockdown both in MKN-45 and AGS cell lines. A CCK-8 assay revealed that silencing *DLGAP1-AS1* could significantly inhibit gastric cancer cell proliferation (Figure 5G, 5H). And the raw data of our results are shown in Supplementary Table 4. Furthermore, a migration and invasion assay indicated that *DLGAP1-AS1* knockdown also plays an inhibitor role in GC migration and invasion (Supplementary Figure 7A–7D). The *DLGAP1-AS1/miR-203a-3p/THBS2* ceRNA pathway and its potential roles in the progression of GC is illustrated in schematic representations in Figure 6. Altogether, we have described a ceRNA network that could shed light on the oncogenesis of GC and may contain future diagnostic markers and therapeutic targets.

## DISCUSSION

Some evidence has indicated that lncRNAs play regulatory roles in the oncogenesis and tumor progression of various cancers [12]. Recent research has suggested that lncRNAs interact with miRNA and regulate downstream mRNA expression [7]. In GC, lncRNAs, miRNA and mRNA function as a unit, not simply through one-to-one interactions. For instance, Huang et al. reported that lncRNA *IGF2-AS* was involved in encouraging tumor growth and invasion in GC through the *IGF2-AS/miR-503/SHOX*2 ceRNA network [13]. Xiao et al. indicated that *TRPM2-AS* functioned in a ceRNA network and negatively regulated *miR-612* expression, thereby leading to GC progression and radio resistance by disrupting the expression of IGF2BP1 and FOXM1 [14]. Wei et al. demonstrated that the *CTC-497E21.4/miR-22-3p/NET1* ceRNA network played positive roles in GC progression via the RhoA signaling pathway [15].

In the present study, we successfully identified a promising *DLGAP1-AS1/miR-203a-3p/THBS2*ceRNA network involved in GC progression through integrated bioinformatic analysis and verification assays. First, we detected 84 upregulated DEGs and 106 downregulated DEGs that were commonly expressed in the training group. GO term and KEGG pathway functional analysis revealed that those DEGs were significantly enriched in the cancer-related process. Then, those DEGs were visualized in the PPI network and selected as hub genes according to their node degrees, which were calculated by the cytoHubba tool. The expression role and survival value of the top 10 hub genes were validated using GEPIA and KM plotter databases. Eight qualified genes met the criteria of expression validation and survival analyses. Notably, their oncogenic roles were also detected in GC progression. For example, the high expression of *THBS2* and *COL1A2* facilitated GC proliferation and invasion but inhibited tumor apoptosis through the PI3K-Akt signaling pathway [16]. Overexpression of *COL1A1* was increased cancer invasion and was directly regulated by *let-7i* miRNA [17]. BGN induced phosphorylation of FAK and Paxillin in GC metastasis, thereby activating the FAK signaling pathway [18].

Additionally, the upstream miRNAs of hub genes were predicted and validated by the relational databases mentioned above. Seven qualified miRNAs were selected as key miRNAs. Some of the miRNAs were involved in the development of GC. For instance, Yang et al. demonstrate that *miR-203a* served as a tumor suppressor and was able to inhibit the proliferation of GC cells by direct bonding with *E2F3* [19]. Li et al. showed that *miR-26a-5p* could impede proliferation and the invasion of GC cells by targeting *COL10A1* [20] Zhang et al. demonstrated that *miR-204a-5p* functioned as an oncogene by targeting *USP47* and *RAB22A* in stomach cancer [21].

Next, we identified the upstream lncRNAs of key miRNAs. Four lncRNAs conformed to expression and prognostic standards. *DLGAP1-AS1* facilitated HCC proliferation and progression via the *miR-486-5p/H3F3B* axis [22]. The elevated expression of *PVT1*has been significantly correlated with poor prognosis in multiple types of cancer [23, 24], including GC [25]. Some interactions within the ceRNA network have been previously identified. These include the *DLGAP-1-AS1/miR-26a-5p* axis in GC and the *PVT1/miR-203a* axis in multiple myeloma [26, 27]. Finally, we established a novel *DLGAP1-AS1/* *miRNA-203a-3p/THBS2* ceRNA network that satisfies the conditions of the ceRNA hypothesis.

Bioinformatic analysis had previously been performed on a ceRNA network linked with GC [28, 29]. However, few studies concentrated on the prognostic value of the ceRNA axis using multi-omics analysis combined with experimental tests. Furthermore, to our knowledge, this is the first study of GC that constructed a ceRNA network in the order of the mRNA-miRNA-lncRNA pattern. There are some limitations to our study. First, we did not stratify the samples based on their clinical characteristics, such as sex. A recent study has indicated that incorporating sex as a biological variable is reveals new information about cancer mechanisms [30]. Second, we verified the expression and prognostic value using online databases rather than the date from clinical samples. To offset this limitation, we constructed the ceRNA axis through comprehensive analysis and validated the data under the same conditions.

In summary, by multi-omics analysis and experimental verification, we successfully constructed a *DLGAP1-AS1/miR-203a-3p/THBS2* ceRNA regulatory network in which all RNAs are correlated with the prognosis of GC patients. In addition to the prognostic value of this network, it also provides some key clues for future molecular mechanism explorations.

## MATERIALS AND METHODS

### Data selection

To identify compressive gene expression patterns in GC samples versus normal samples, we obtained the mRNA microarray profiles from GEO (www.ncbi.nlm.nih.gov/geo/) datasets. Only datasets consisting of at least 20 samples of both GC and normal tissues were collected. Eventually, 6 GEO datasets were selected for subsequent analyses. To increase the reliability of our research, GSE54129, GSE29272 and GSE13911 were chosen as a training group, whereas GSE27342, GSE37023 and GSE65801 were selected as a validation group. Furthermore, the TCGA database was integrated into the training group to enhance the reliability of our results.

### Identification of DEGs and functional annotation analysis

Raw RNA-seq data were downloaded from the UCSC TCGA website. These datasets comprised 380 GC samples and 37 normal samples (https://xena.ucsc.edu/public/) [31]. The raw data were annotated by relational platforms and standardized by the method of log_2_(x+1). Datasets were normalized using the “normalize between array” function of the LIMMA package from R Software (version 3.6.1) [32]. The package was used to screen differential mRNAs with thresholds of |log2FC| > 1 and a *P* value < 0.05. The commonly expressed mRNAs in the training group were defined as the DEGs and divided into upregulated DEGs and downregulated DEGs. These data were visualized through Venn diagrams using VENNY 2.1.0 (https://bioinfogp.cnb.csic.es/tools/venny/index.html) [33].

To elucidate the potential functions of the DEGs, we performed the GO functional enrichment analysis and KEGG pathway analysis via DAVIDv6.8 (https://david.ncifcrf.gov/) [34] and KOBAS 3.0 (http://kobas.cbi.pku.edu.cn/) software [35]. The top 10 enriched GO terms and KEGG pathways were visualized by the “ggplot2” package with cut-off criteria of *P*< 0.05 [36].

### Hub genes identification and validation

The STRING v11.0 database (https://string-db.org/) [37] revealed a PPI network for DEGs with a combined confidence score ≥0.4. Next, we utilized cytoHubba, an app in Cytoscape software (Version 3.7.2) [38], to identify the top 20 hub genes according to their connection degree within the PPI network. We validated the expression levels of hub genes using the GEPIA database to analyze 408 GC samples and 211 normal controls from TCGA and Genotype-Tissue Expression GTEx data (http://gepia.cancer-pku.cn/index.html) [39]. The threshold value was set as |logFC| >1 and *P*< 0.01. Ultimately, the prognostic roles of key genes expressed in 436 GC samples were evaluated through the KM plotter (http://kmplot.com) [40]. The hazard ratio with a 95% confidence interval and log-rank *P* value was generated online. *P*< 0.05 was viewed as a statistically significant.

### Prediction of upstream miRNA and lncRNA

To obtain comprehensive and reliable prediction results, we used the miRecords database that integrates the 11miRNA target prediction tools to predict the upstream miRNAs of hub genes [41]. The tools were DIANA-micro T, MicroInspector, miRanda, MirTarget2, miTarget, NBmiRTar, PicTar, PITA, RNA22, RNAhybrid and TargetScan. Only miRNAs that appeared at least 3 times were considered as validation miRNAs. The miRNet, was employed to find potential lncRNAs that would bind to validation miRNAs (https://www.mirnet.ca/miRNet/) [42]. GEPIA and KM plot databases verified the effectiveness of potential lncRNAs.

### Correlation analysis and experimental verification

We used the starBase platform, a tool that evaluates potential RNA-RNA interactions [43], to validate predicted miRNA and lncRNA. We also assessed the entire interrelation among the lncRNA, miRNA and genes via the starBase platform.

We validated the regulatory role of the ceRNA network in vitro assays. Normal gastric cells (GES-1) and Human GC cell lines (MKN-45, AGS, HGC-27 and MGC-803) were obtained from the cell bank of the Chinese Scientific Academy (Shanghai, China). All cells were incubated in RPMI-1640 (Gibco, Life Technologies, CA, USA), supplemented with 10% fetal bovine serum (Certified, US origin) and incubated at 37°C and 5% CO_2_.

Three siRNAs (*si-DLGAP1-AS1*#1: 5’-GCU AUA UGU CUG GUA AAC AGA-3’, *si-DLGAP1-AS1*#2: 5’-CAG AAU AAA UAG UAC UUG AGC-3’ and*si-DLGAP1-AS1*#3: 5’-GCU GCU AUA UGU CUG GUA AAC-3’) and negative control siRNA were purchased from Riobio Company (Guangzhou, China) and separately transfected into MKN-45 and AGS cells using Lipofectamine 3000 (Invitrogen, USA). Then, the qRT-PCR assay was performed to evaluate knockdown efficiency and detect the relative expression of *DLGAP1-AS1, miR-203a-3p* and *THBS2* through the SYBR-green method. Primer sequences were also synthesized by Riobio as shown as follows: *DLGAP1-AS1*:5’-GGG GCA GGA GTA AAG TGG AC-3’ (forward), 5’-CCA GAC ATA TAG CAG CCG GG-3’ (reverse); *miR-203a-3p*: 5’-CAC CAT AAA GAC AGG AAC CTG-3’ (forward), 5’-GGA GGT GCC ATC AAT ACC TGC-3’ (reverse); *THBS2*: 5’-TTA TGG CGT TGC ATC CAG GT-3’ (forward), 5’-GTG GTG CAG AGG AGA TGT GT-3’ (reverse); and *GAPDH*:5’-GAT TTG GTC GTA TTG GGC GC-3’ (forward), 5’-GCG CCC AAT ACG ACC AAA TC-3’ (reverse). The relative expression levels were quantified using the 2^−ΔΔCt^ method.

Cell viability detection was measured using the Cell Counting Kit-8 (CCK-8) assay according to instructions (Dojindo, Kumamoto, Japan). In brief, we incubated transfected cells in 96-well plates and then measured their viability with the CCK-8 kit at 0, 24, 48, 72 and 96 hours. The absorbance was detected at 450 nm with a microplate reader (BioTek, VT, USA). The result was repeated 3 times.

Wound healing, migration and invasion assays were performed. For the wound healing assay, the movement of cells was measured in a scrape, which was made with a sterile 200 μL pipette tip, and the spread of the wound closure was observed after 24 hours. The scratched areas at 0 and 24 hours were photographed at 100x magnification using an inverted microscope (Nikon DS-Ri2, Japan). For migration assay, transwell chambers with 8-μm porous membranes (Corning, NY, USA) were used. 80,000 cells were seeded onto the upper chamber with 200 μL serum-free medium, and 800 μL medium containing 10% FBS was added to the bottom chambers as a chemo-attractant. Following 24 hours of incubation, cells that did not migrate to the bottom chambers were removed from the top side with a cotton swab. The invasion assay was performed in a similar procedure except the upper chambers were coated with Matrigel (BD Biosciences), which was diluted at 1:10 with serum-free medium. Cells traversing the membranes were fixed and stained with a Hematoxylin-Eosin Staining Kit (Solarbio, Beijing, China) according to the manufacturer’s instruction. Cell images were captured using a microscope (Nikon E800) at 200x magnification and 5 random fields per insert were counted. Results were presented as cells migrated per field.

### Statistical analysis

Most of the statistical analyses was conducted using the R Software and the other bioinformatic tools mentioned above. GraphPad Prism 7.0 (IBM, New York City, NY, USA) software was also utilized to analyze data. The two-tailed Student’s *t*-test was applied to analyze the relative expression levels of mRNA, miRNA and lncRNA. Correlations between RNA expression were evaluated through Pearson correlation analysis. A *P* value<0.05 was considered statistically significant, which is presented in figures according to *P< 0.05, **P< 0.01 and ***P< 0.001.

## Supplementary Material

Supplementary Figures

Supplementary Table 1

Supplementary Table 2

Supplementary Table 3

Supplementary Table 4
