# Supplementary material for: Identification of a competing endogenous RNA axis related to gastric cancer
Source: Aging (Albany NY). 2020 Oct 20;12(20):20540–60. doi: 10.18632/aging.103926 (PMC7655175; doi:10.18632/aging.103926)
Supplement: Supplementary Figures [file aging-12-103926-s001..pdf]

SUPPLEMENTARY FIGURES

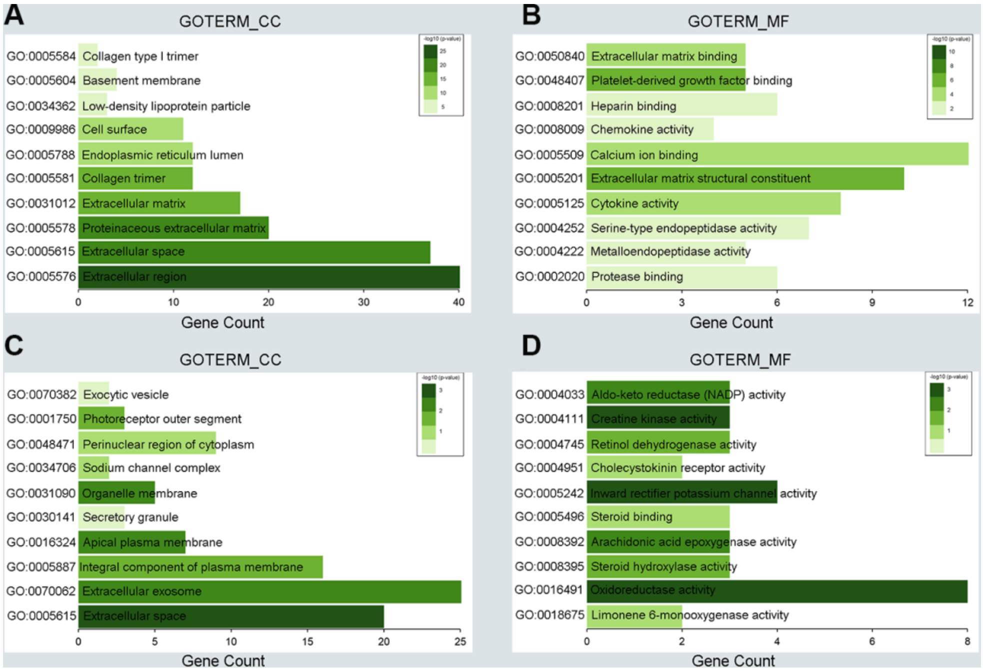

Supplementary Figure 1. Functional enrichment analysis for the significant DEGs. (**A**, **C**) The top ten enriched cellular components of the significantly upregulated DEGs and downregulated DEGs respectively. (**B**, **D**) The top ten enriched molecular function of the significantly upregulated DEGs and downregulated DEGs respectively.

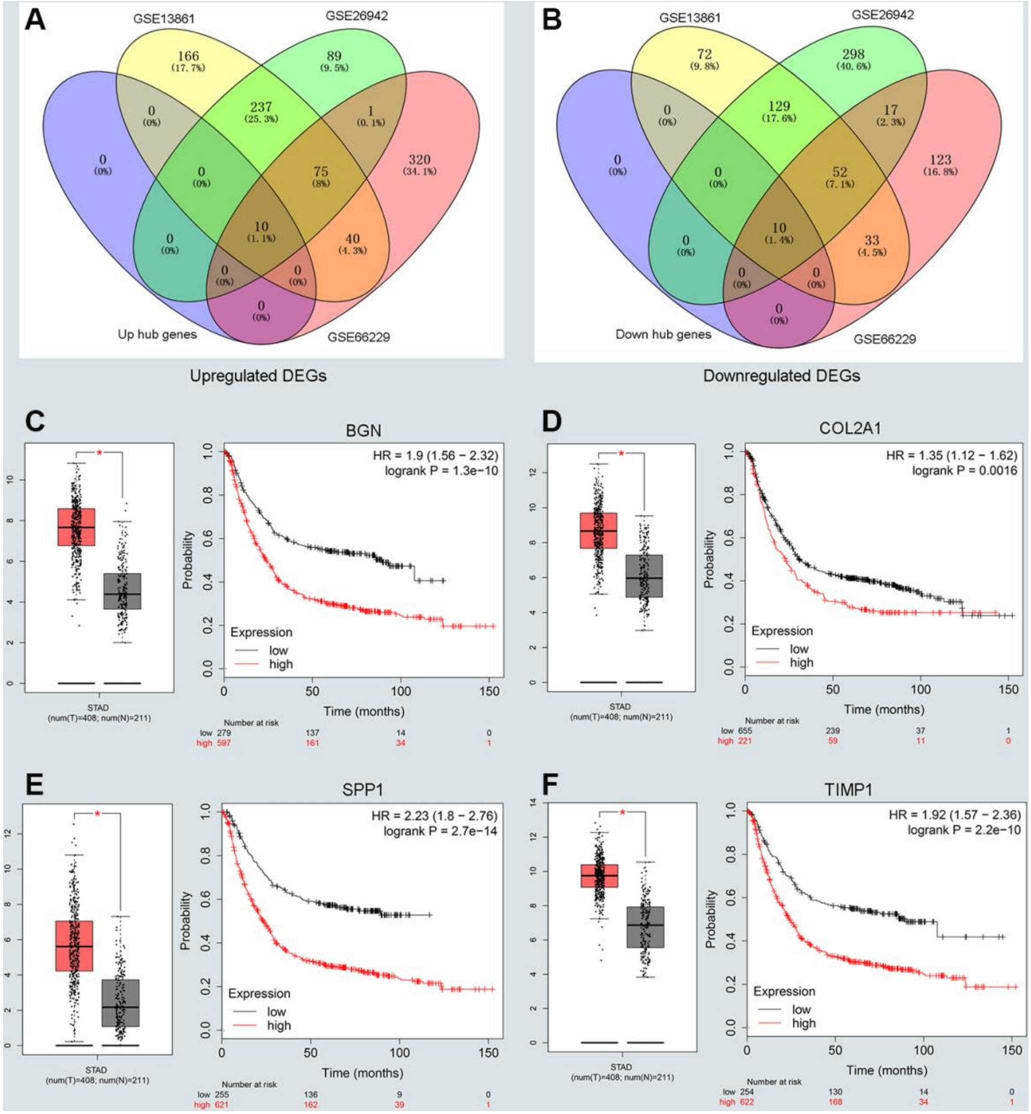

Supplementary Figure 2. Verifying the distribution of hub genes in the validation group and identifying key genes in GC. (**A**, **B**) The intersection of the top ten key hub genes in the validation group (GSE27342, GSE37023, and GSE65801). (**C**–**F**) Validating expression roles and prognosis values of key genes in hub genes using GEPIA and Kaplan–Meier plotter databases.

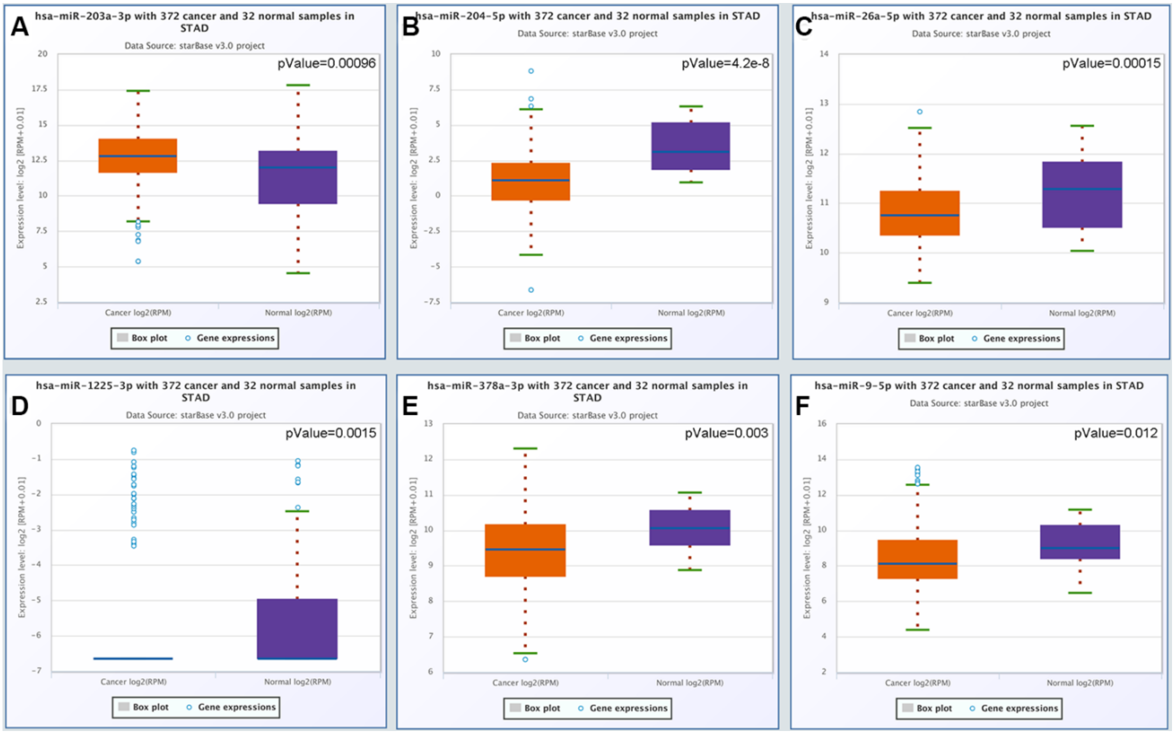

Supplementary Figure 3. Screening and validating the expression roles of key miRNAs in GC. (**A**–**F**) Validating expression roles of key miRNAs using GEPIA databases.

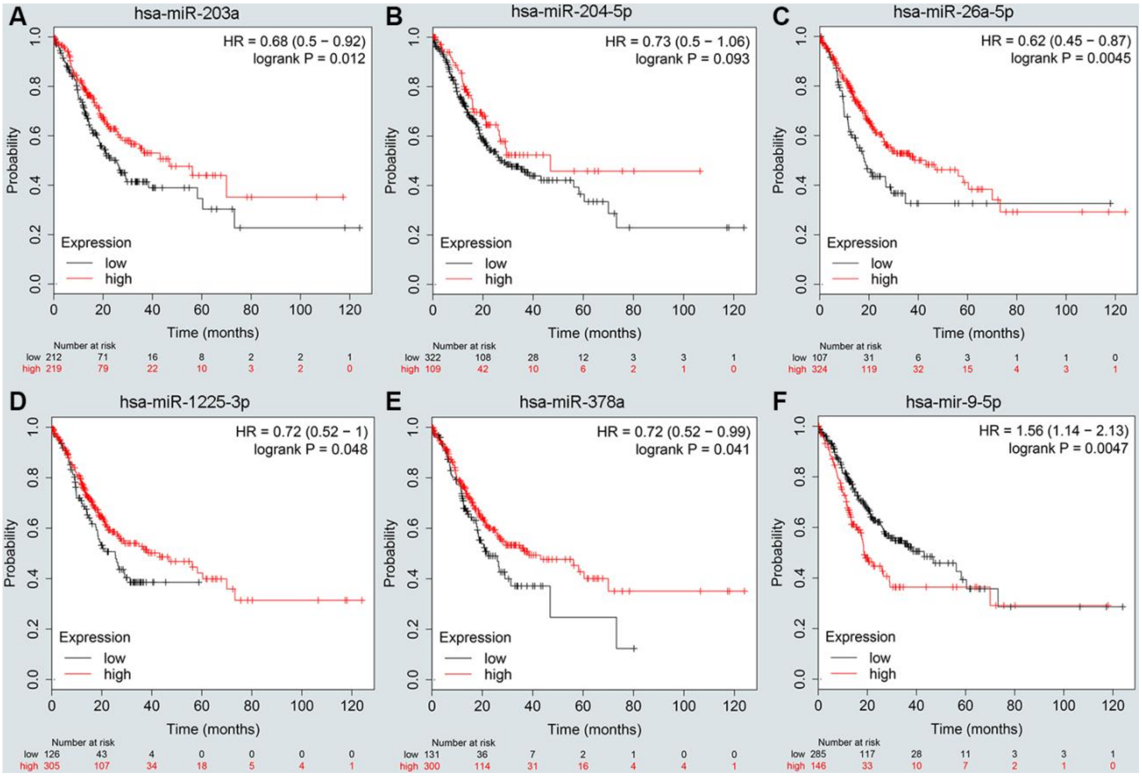

Supplementary Figure 4. Screening and validating the prognostic values of key miRNAs in GC. (**A**–**F**) Validating prognosis values of key miRNAs using Kaplan–Meier plotter databases.

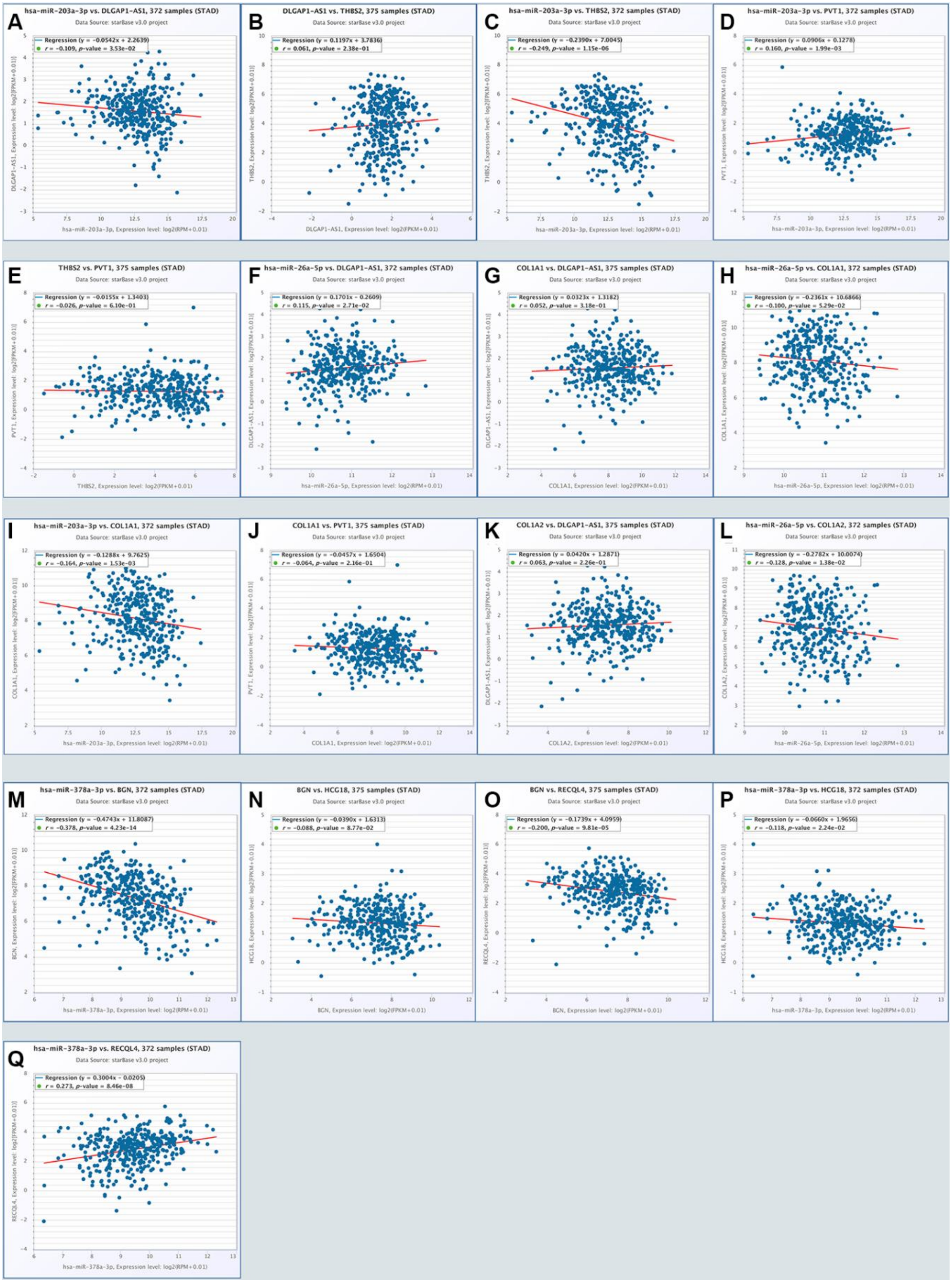

Supplementary Figure 5. Identifying the qualified ceRNA network through correlation analysis. Only DLGAP1-AS1/ miR-203a-3p/THBS2 ceRNA axis met the correlation analysis according to the ceRNA hypothesis (**A**–**C**). Other ceRNA networks failed the criteria that lncRNAs positively associated with mRNAs while miRNAs negative related to lncRNAs and mRNAs (**D**–**Q**).

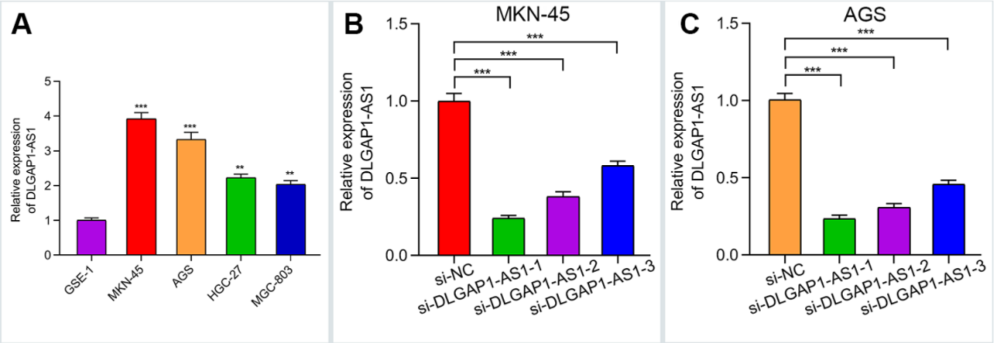

Supplementary Figure 6. Detecting the expressed of DLGAP1-AS1 in GC cell lines and assessing knockdown efficiency. (**A**) The expression of DLGAP1-AS1 in GC cell lines (MKN-45, AGS, HGC-27, and MGC-803) and a normal cell line GSE-1 detected by RT-qPCR. (**B**, **C**) Knockdown efficiency of DLGAP1-AS1 in MKN-45 and AGS cells.

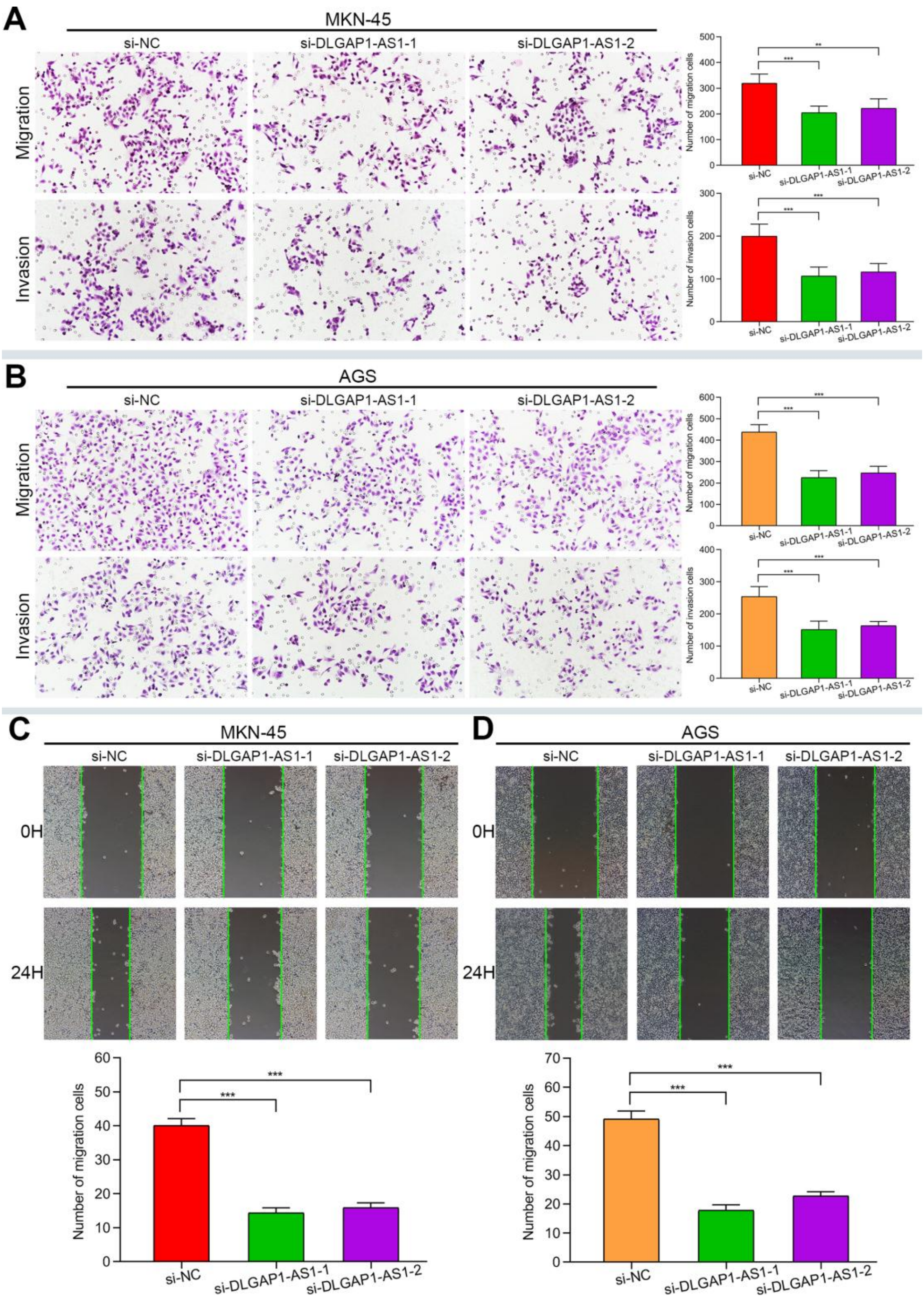

Supplementary Figure 7. Cell migration and invasion assays in MKN-45 and AGS cells. (**A**) Cell migration and invasion assays in si-NC, si-DLGAP1-AS1-1 and si-DLGAP1-AS1-2 transfected MKN-45 cells. (**B**) Cell migration and invasion assays in si-NC, si-DLGAP1-AS1-1 and si-DLGAP1-AS1-2 transfected AGS cells. (**C**) Wound-healing assay assays in si-NC, si-DLGAP1-AS1-1 and si-DLGAP1-AS1-2 transfected MKN-45 cells. (**D**) Wound-healing assay assays in si-NC, si-DLGAP1-AS1-1 and si-DLGAP1-AS1-2 transfected MKN-45 cells. Data are presented as means of three different experiments. Bars indicate ± SE. * P<0.05, **P<0.01, and ***P<0.001 compared with control groups.
