# Supplementary material for: Identification of a competing endogenous RNA axis related to gastric cancer
Source: Aging (Albany NY). 2020 Oct 20;12(20):20540–60. doi: 10.18632/aging.103926 (PMC7655175; doi:10.18632/aging.103926)
Supplement: Supplementary Table 1 [file aging-12-103926-s002..pdf]

SUPPLEMENTARY TABLE

Supplementary Table 1. The commonly upregulated or downregulated genes in GSE54129, GSE29272 and GSE13911 datasets and TCGA databases.

Upregulated genes	Downregulated genes
APOE	ACER2
ASPN	ADH1C
BGN	ADH7
CDH11	ADHFE1
CDH3	ADRB2
CEMIP	AKR1B10
CHI3L1	AKR1B15
CLDN1	AKR1C1
CLEC5A	AKR7A3
COL10A1	ALDH3A1
COL11A1	ALDH6A1
COL12A1	ALDOB
COL1A1	AMPD1
COL1A2	ANXA10
COL3A1	ATP4A
COL4A1	ATP4B
COL5A2	B3GNT6
COL8A1	BHLHA15
COMP	CA2
CPXM1	CAPN9
CST1	CCKAR
CST2	CCKBR
CTHRC1	CHGA
CXCL1	CKB
CXCL10	CKM
CXCL8	CKMT2
CXCL9	CLIC6
DTL	CPB1
ECT2	CTSE
F2RL2	CYP2C18
FAP	CYP2C19
FKBP10	CYP2C9
FNDC1	DPT
HOXA10	DUOX1
HOXA13	EPN3
HOXC10	ESRRG
HOXC6	ETNPPL
IFITM1	FAM46C
IGF2BP3	FBP2
INHBA	FCGBP
ITGBL1	FMO5
KLK6	GATA5
LAIR2	GCNT4
LIF	GHRL
LIPG	GIF
LOX	GKN1
LY6E	GKN2
MAGEA6	GPER1
MFAP2	GRIA4
MMP11	GSTA1
MMP12	GSTA3
MMP3	GUCA2B
MMP7	HDC
MSR1	HOMER2
NOX4	IGJ
OLFM4	IRX3
OLFML2B	KCNE2
OLR1	KCNJ13
P4HA3	KCNJ15
PDGFRB	KCNJ16

PLA2G2A	KLF4
PLA2G7	KRT20
PLAU	LDHD
PMEPA1	LTF
PRRX1	MAMDC2
RARRES1	MAOA
SALL4	MT1E
SERPINE1	MT1G
SERPINH1	MT1H
SFRP4	MT1M
SNX10	MT1X
SPARC	MUC6
SPP1	MYRIP
SULF1	NKX6-2
THBS2	NR0B2
THY1	PDIA2
TIMP1	PGA4
TNFAIP6	PGC
TNFRSF11B	PIGR
TNFSF4	RAB27A
TREM1	RDH12
TREM2	REG3A
VCAN	SCGB2A1
WNT2	SCNN1B
	SCNN1G
	SH3GL2
	SIDT2
	SLC26A7
	SLC5A5
	SMPD3
	SOX21
	SPTSSB
	SST
	SSTR1
	SULT2A1
	SYTL5
	TCN1
	TFF1
	TFF2
	TMED6
	TNFRSF17
	UGT2B15
	UPK1B
	VSIG1
	VSIG2
	VSTM2A
