# Supplementary material for: Identification of a competing endogenous RNA axis related to gastric cancer
Source: Aging (Albany NY). 2020 Oct 20;12(20):20540–60. doi: 10.18632/aging.103926 (PMC7655175; doi:10.18632/aging.103926)
Supplement: Supplementary Table 2 [file aging-12-103926-s003..docx]

**Supplementary Table 2. The mRNA-miRNA pairs predicted by the miRecords database.**

| mRNA | miRNA |
| --- | --- |
| THBS2 | hsa-miR-182 |
| THBS2 | hsa-miR-203-3p |
| THBS2 | hsa-miR-495 |
| THBS2 | hsa-miR-802 |
| THBS2 | hsa-miR-607 |
| THBS2 | hsa-miR-598 |
| THBS2 | hsa-miR-135b |
| THBS2 | hsa-miR-519c-3p |
| THBS2 | hsa-miR-519b-3p |
| THBS2 | hsa-miR-519a |
| THBS2 | hsa-miR-660 |
| THBS2 | hsa-miR-668 |
| THBS2 | hsa-miR-380 |
| THBS2 | hsa-miR-891b |
| THBS2 | hsa-miR-768-3p |
| THBS2 | hsa-miR-587 |
| THBS2 | hsa-miR-656 |
| THBS2 | hsa-miR-576-5p |
| THBS2 | hsa-miR-559 |
| THBS2 | hsa-miR-545 |
| THBS2 | hsa-miR-591 |
| THBS2 | hsa-miR-513a-5p |
| THBS2 | hsa-miR-499-5p |
| THBS2 | hsa-miR-522 |
| THBS2 | hsa-miR-616 |
| THBS2 | hsa-miR-618 |
| THBS2 | hsa-miR-630 |
| THBS2 | hsa-miR-30a |
| THBS2 | hsa-miR-135a |
| THBS2 | hsa-miR-30e |
| THBS2 | hsa-miR-30b |
| THBS2 | hsa-miR-511 |
| THBS2 | hsa-miR-29b |
| THBS2 | hsa-miR-30d |
| THBS2 | hsa-miR-30c |
| THBS2 | hsa-miR-1 |
| THBS2 | hsa-miR-144 |
| THBS2 | hsa-miR-204-5p |
| THBS2 | hsa-miR-29c |
| THBS2 | hsa-miR-490-3p |
| THBS2 | hsa-miR-497 |
| COL1A1 | hsa-miR-29c |
| COL1A1 | hsa-let-7i |
| COL1A1 | hsa-let-7g |
| COL1A1 | hsa-miR-218 |
| COL1A1 | hsa-let-7e |
| COL1A1 | hsa-let-7d |
| COL1A1 | hsa-let-7c |
| COL1A1 | hsa-let-7b |
| COL1A1 | hsa-let-7a |
| COL1A1 | hsa-miR-98 |
| COL1A1 | hsa-miR-196b |
| COL1A1 | hsa-miR-196a |
| COL1A1 | hsa-miR-29a |
| COL1A1 | hsa-let-7f |
| COL1A1 | hsa-miR-328 |
| COL1A1 | hsa-miR-527 |
| COL1A1 | hsa-miR-765 |
| COL1A1 | hsa-miR-661 |
| COL1A1 | hsa-miR-622 |
| COL1A1 | hsa-miR-548c-3p |
| COL1A1 | hsa-miR-514 |
| COL1A1 | hsa-miR-577 |
| COL1A1 | hsa-miR-185 |
| COL1A1 | hsa-miR-29b |
| COL1A1 | hsa-miR-150 |
| COL1A1 | hsa-miR-338-3p |
| COL1A1 | hsa-miR-26a-5p |
| COL1A1 | hsa-miR-203a-3p |
| COL1A1 | hsa-miR-195 |
| COL1A1 | hsa-miR-339-5p |
| COL1A1 | hsa-miR-491-5p |
| COL1A1 | hsa-miR-497 |
| COL1A1 | hsa-miR-490-5p |
| COL1A1 | hsa-miR-1225-3p |
| COL1A2 | hsa-miR-29c |
| COL1A2 | hsa-miR-29a |
| COL1A2 | hsa-miR-196b |
| COL1A2 | hsa-miR-25 |
| COL1A2 | hsa-miR-367 |
| COL1A2 | hsa-miR-92b |
| COL1A2 | hsa-miR-98 |
| COL1A2 | hsa-miR-563 |
| COL1A2 | hsa-let-7e |
| COL1A2 | hsa-miR-26a-5p |
| COL1A2 | hsa-miR-29b |
| COL1A2 | hsa-let-7a |
| COL1A2 | hsa-let-7b |
| COL1A2 | hsa-let-7c |
| COL1A2 | hsa-let-7f |
| COL1A2 | hsa-let-7d |
| COL1A2 | hsa-miR-584 |
| COL1A2 | hsa-miR-92a |
| COL1A2 | hsa-miR-32 |
| COL1A2 | hsa-miR-642 |
| COL1A2 | hsa-miR-298 |
| COL1A2 | hsa-miR-768-3p |
| COL1A2 | hsa-miR-767-5p |
| COL1A2 | hsa-miR-625 |
| COL1A2 | hsa-miR-196a |
| COL1A2 | hsa-miR-587 |
| COL1A2 | hsa-miR-105 |
| COL1A2 | hsa-miR-552 |
| COL1A2 | hsa-miR-342-3p |
| COL1A2 | hsa-miR-7 |
| COL1A2 | hsa-let-7i |
| COL1A2 | hsa-miR-26b |
| COL1A2 | hsa-miR-186 |
| COL1A2 | hsa-let-7g |
| COL1A2 | hsa-miR-363 |
| SPP1 | hsa-miR-181a |
| SPP1 | hsa-miR-181b |
| SPP1 | hsa-miR-181c |
| SPP1 | hsa-miR-127-5p |
| SPP1 | hsa-miR-518a-5p |
| SPP1 | hsa-miR-548d-3p |
| SPP1 | hsa-miR-570 |
| SPP1 | hsa-miR-888 |
| SPP1 | hsa-miR-181d |
| SPP1 | hsa-miR-130b |
| SPP1 | hsa-miR-130a |
| SPP1 | hsa-miR-154 |
| BGN | hsa-miR-608 |
| BGN | hsa-miR-552 |
| BGN | hsa-miR-647 |
| BGN | hsa-miR-502-5p |
| BGN | hsa-miR-765 |
| BGN | hsa-miR-423-5p |
| BGN | hsa-miR-149 |
| BGN | hsa-miR-326 |
| BGN | hsa-miR-383 |
| BGN | hsa-miR-378a |
| BGN | hsa-miR-491-5p |
| TIMP1 | hsa-miR-147b |
| TIMP1 | hsa-miR-657 |
| TIMP1 | hsa-miR-1231 |
| TFF2 | hsa-miR-876-5p |
| TFF2 | hsa-miR-573 |
| TFF2 | hsa-miR-524-3p |
| TFF2 | hsa-miR-9 |
| TFF2 | hsa-miR-202 |
| SST | hsa-miR-768-5p |
| SST | hsa-miR-765 |
| SST | hsa-miR-582-5p |
| SST | hsa-miR-432 |
| SST | hsa-miR-23a |
| SST | hsa-miR-383 |
| SST | hsa-miR-190b |
| SST | hsa-miR-197 |
