# Supplementary material for: Identification of a competing endogenous RNA axis related to gastric cancer
Source: Aging (Albany NY). 2020 Oct 20;12(20):20540–60. doi: 10.18632/aging.103926 (PMC7655175; doi:10.18632/aging.103926)
Supplement: Supplementary Table 3 [file aging-12-103926-s004..docx]

**Supplementary Table 3. The miRNA-lncRNA pairs predicted by the miRNet database.**

| miRNA | lncRNA |
| --- | --- |
| hsa-mir-203a | NEFL |
| hsa-mir-203a | PVT1 |
| hsa-mir-203a | XIST |
| hsa-mir-203a | ZNF518A |
| hsa-mir-203a | HCP5 |
| hsa-mir-203a | C14orf169 |
| hsa-mir-203a | LINC00263 |
| hsa-mir-203a | DGKK |
| hsa-mir-203a | FBXL19-AS1 |
| hsa-mir-203a | MALAT1 |
| hsa-mir-203a | MIR17HG |
| hsa-mir-203a | HCG11 |
| hsa-mir-203a | OIP5-AS1 |
| hsa-mir-203a | AC005082.12 |
| hsa-mir-203a | AC009948.5 |
| hsa-mir-203a | AC093375.1 |
| hsa-mir-203a | ADIRF-AS1 |
| hsa-mir-203a | AP003774.1 |
| hsa-mir-203a | CTB-89H12.4 |
| hsa-mir-203a | DLGAP1-AS1 |
| hsa-mir-203a | hsa-mir-6080 |
| hsa-mir-203a | KB-431C1.4 |
| hsa-mir-203a | KIAA1984-AS1 |
| hsa-mir-203a | LINC00657 |
| hsa-mir-203a | LINC00667 |
| hsa-mir-203a | RP11-1094M14.11 |
| hsa-mir-203a | RP11-1149O23.3 |
| hsa-mir-203a | RP11-145M9.4 |
| hsa-mir-203a | RP11-156E6.1 |
| hsa-mir-203a | RP11-159D12.9 |
| hsa-mir-203a | RP11-196G18.22 |
| hsa-mir-203a | RP11-227G15.3 |
| hsa-mir-203a | RP11-311C24.1 |
| hsa-mir-203a | RP11-342K6.1 |
| hsa-mir-203a | RP11-344E13.3 |
| hsa-mir-203a | RP11-361F15.2 |
| hsa-mir-203a | RP11-372K14.2 |
| hsa-mir-203a | RP11-446J8.1 |
| hsa-mir-203a | RP11-473I1.10 |
| hsa-mir-203a | RP11-49C24.1 |
| hsa-mir-203a | RP11-51M18.1 |
| hsa-mir-203a | RP11-5L12.1 |
| hsa-mir-203a | RP11-65L3.2 |
| hsa-mir-203a | RP11-672F9.1 |
| hsa-mir-203a | RP11-902B17.1 |
| hsa-mir-203a | RP4-545L17.7 |
| hsa-mir-203a | RP4-714D9.2 |
| hsa-mir-203a | RP5-1061H20.4 |
| hsa-mir-204-5p | SLC37A4 |
| hsa-mir-204-5p | XIST |
| hsa-mir-204-5p | KCNQ1OT1 |
| hsa-mir-204-5p | DCP1A |
| hsa-mir-204-5p | LINC00472 |
| hsa-mir-204-5p | PPP1R9B |
| hsa-mir-204-5p | MCM3AP-AS1 |
| hsa-mir-204-5p | NEAT1 |
| hsa-mir-204-5p | HNRNPU-AS1 |
| hsa-mir-204-5p | MALAT1 |
| hsa-mir-204-5p | CTA-204B4.6 |
| hsa-mir-204-5p | CTB-96E2.3 |
| hsa-mir-204-5p | CTC-281B15.1 |
| hsa-mir-204-5p | DHRS4-AS1 |
| hsa-mir-204-5p | RP11-156E6.1 |
| hsa-mir-204-5p | RP11-159D12.2 |
| hsa-mir-204-5p | RP11-220I1.1 |
| hsa-mir-204-5p | RP11-252A24.7 |
| hsa-mir-204-5p | RP11-258C19.5 |
| hsa-mir-204-5p | RP11-27M15.1 |
| hsa-mir-204-5p | RP11-435O5.2 |
| hsa-mir-204-5p | RP11-658F2.8 |
| hsa-mir-204-5p | RP11-746E8.1 |
| hsa-mir-204-5p | RP1-37E16.12 |
| hsa-mir-204-5p | RP3-331H24.5 |
| hsa-mir-204-5p | RP3-368A4.5 |
| hsa-mir-204-5p | RP4-773N10.5 |
| hsa-mir-204-5p | RP5-1024G6.8 |
| hsa-mir-204-5p | RP6-24A23.7 |
| hsa-mir-26a-5p | NEFL |
| hsa-mir-26a-5p | SCAMP1 |
| hsa-mir-26a-5p | CWC15 |
| hsa-mir-26a-5p | GAS5 |
| hsa-mir-26a-5p | ZNF718 |
| hsa-mir-26a-5p | LINC00205 |
| hsa-mir-26a-5p | HNRNPU-AS1 |
| hsa-mir-26a-5p | DLX6-AS1 |
| hsa-mir-26a-5p | MALAT1 |
| hsa-mir-26a-5p | SNHG5 |
| hsa-mir-26a-5p | HCG11 |
| hsa-mir-26a-5p | SNHG6 |
| hsa-mir-26a-5p | OIP5-AS1 |
| hsa-mir-26a-5p | WASIR2 |
| hsa-mir-26a-5p | LINC00240 |
| hsa-mir-26a-5p | MIR4720 |
| hsa-mir-26a-5p | AC005082.12 |
| hsa-mir-26a-5p | AC058791.2 |
| hsa-mir-26a-5p | AL589743.1 |
| hsa-mir-26a-5p | AP000525.9 |
| hsa-mir-26a-5p | CTD-2270L9.5 |
| hsa-mir-26a-5p | CTD-2314B22.3 |
| hsa-mir-26a-5p | CTD-2555O16.2 |
| hsa-mir-26a-5p | DLGAP1-AS1 |
| hsa-mir-26a-5p | hsa-mir-6080 |
| hsa-mir-26a-5p | LINC00610 |
| hsa-mir-26a-5p | LINC00657 |
| hsa-mir-26a-5p | LINC00847 |
| hsa-mir-26a-5p | RP11-1006G14.4 |
| hsa-mir-26a-5p | RP11-105N13.4 |
| hsa-mir-26a-5p | RP11-175O19.4 |
| hsa-mir-26a-5p | RP11-276H19.2 |
| hsa-mir-26a-5p | RP11-282O18.3 |
| hsa-mir-26a-5p | RP11-31E23.1 |
| hsa-mir-26a-5p | RP11-396C23.2 |
| hsa-mir-26a-5p | RP11-452F19.3 |
| hsa-mir-26a-5p | RP11-473I1.10 |
| hsa-mir-26a-5p | RP11-478C19.2 |
| hsa-mir-26a-5p | RP11-492E3.2 |
| hsa-mir-26a-5p | RP11-706J10.1 |
| hsa-mir-26a-5p | RP11-738E22.2 |
| hsa-mir-26a-5p | RP11-78O7.2 |
| hsa-mir-26a-5p | RP5-1172N10.3 |
| hsa-mir-26a-5p | RP5-1172N10.4 |
| hsa-mir-26a-5p | RP6-24A23.7 |
| hsa-mir-26a-5p | SETD5-AS1 |
| hsa-mir-26a-5p | XXbac-B461K10.4 |
| hsa-mir-26a-5p | XXbac-BPG32J3.20 |
| hsa-mir-339-5p | JRK |
| hsa-mir-339-5p | RECQL4 |
| hsa-mir-339-5p | KCNQ1OT1 |
| hsa-mir-339-5p | DCP1A |
| hsa-mir-339-5p | MAFG-AS1 |
| hsa-mir-339-5p | LINC00094 |
| hsa-mir-339-5p | H19 |
| hsa-mir-339-5p | NEAT1 |
| hsa-mir-339-5p | CDKN2B-AS1 |
| hsa-mir-339-5p | CTC-444N24.8 |
| hsa-mir-339-5p | CTD-2270L9.5 |
| hsa-mir-339-5p | hsa-mir-125a |
| hsa-mir-339-5p | hsa-mir-151a |
| hsa-mir-339-5p | RP11-31E23.1 |
| hsa-mir-339-5p | RP11-355O1.11 |
| hsa-mir-339-5p | RP11-498C9.15 |
| hsa-mir-339-5p | RP11-64P12.8 |
| hsa-mir-339-5p | RP3-410C9.1 |
| hsa-mir-339-5p | RP4-773N10.5 |
| hsa-mir-339-5p | RP6-24A23.7 |
| hsa-mir-378a-3p | SLC37A4 |
| hsa-mir-378a-3p | RN7SL1 |
| hsa-mir-378a-3p | RECQL4 |
| hsa-mir-378a-3p | CASP8AP2 |
| hsa-mir-378a-3p | LINC00094 |
| hsa-mir-378a-3p | FBXL19-AS1 |
| hsa-mir-378a-3p | MALAT1 |
| hsa-mir-378a-3p | HCG18 |
| hsa-mir-378a-3p | JPX |
| hsa-mir-378a-3p | OIP5-AS1 |
| hsa-mir-378a-3p | AP006222.2 |
| hsa-mir-378a-3p | CTA-204B4.6 |
| hsa-mir-378a-3p | CTC-444N24.8 |
| hsa-mir-378a-3p | CTC-479C5.17 |
| hsa-mir-378a-3p | LINC00641 |
| hsa-mir-378a-3p | LINC00657 |
| hsa-mir-378a-3p | RP11-159D12.9 |
| hsa-mir-378a-3p | RP11-392M18.5 |
| hsa-mir-378a-3p | RP11-405O10.2 |
| hsa-mir-378a-3p | RP11-519M16.1 |
| hsa-mir-378a-3p | RP11-701H24.5 |
| hsa-mir-378a-3p | RP4-622L5.2 |
| hsa-mir-378a-3p | RP5-837J1.2 |
| hsa-mir-378a-3p | Z97634.5 |
| hsa-mir-9-5p | NEFL |
| hsa-mir-9-5p | XIST |
| hsa-mir-9-5p | MAP3K14 |
| hsa-mir-9-5p | UHRF1 |
| hsa-mir-9-5p | TUG1 |
| hsa-mir-9-5p | ZNRD1-AS1 |
| hsa-mir-9-5p | SNHG7 |
| hsa-mir-9-5p | MAGI1-IT1 |
| hsa-mir-9-5p | NEAT1 |
| hsa-mir-9-5p | HNRNPU-AS1 |
| hsa-mir-9-5p | PCBP1-AS1 |
| hsa-mir-9-5p | HCG18 |
| hsa-mir-9-5p | SENP3-EIF4A1 |
| hsa-mir-9-5p | ZNF252P-AS1 |
| hsa-mir-9-5p | RP6-24A23.7 |
| hsa-mir-9-5p | RP1-37E16.12 |
| hsa-mir-9-5p | RP13-507I23.1 |
| hsa-mir-9-5p | RP11-96D1.10 |
| hsa-mir-9-5p | RP11-793H13.8 |
| hsa-mir-9-5p | RP11-658F2.8 |
| hsa-mir-9-5p | RP11-64K12.2 |
| hsa-mir-9-5p | RP11-622K12.1 |
| hsa-mir-9-5p | RP11-519M16.1 |
| hsa-mir-9-5p | RP11-511P7.2 |
| hsa-mir-9-5p | RP11-473I1.10 |
| hsa-mir-9-5p | RP11-299M14.2 |
| hsa-mir-9-5p | RP11-170L3.8 |
| hsa-mir-9-5p | PTCHD3P1 |
| hsa-mir-9-5p | LINC00665 |
| hsa-mir-9-5p | CTB-25J19.9 |
| hsa-mir-9-5p | AL163636.6 |
| hsa-mir-9-5p | AC093642.3 |
| hsa-mir-9-5p | AC017048.3 |
| hsa-mir-9-5p | AC007246.3 |
| hsa-mir-9-5p | AC005076.5 |
